# Rate of spontaneous voiding recovery after acute urinary retention due to bed rest in the hospital setting in a non-urological population clinical study of the relationship between lower limbs and bladder function

**DOI:** 10.1590/S1677-5538.IBJU.2015.0450

**Published:** 2016

**Authors:** Paulo Rodrigues, Flávio Hering, Eli Cieli, João Carlos Campagnari

**Affiliations:** 1 Clínica de Urologia do Hospital Beneficência Portuguesa de São Paulo - São Paulo, Brasil; 2 Departamento de Urologia, Hospital Santa Helena de São Paulo - São Paulo, Brasil

**Keywords:** Urinary Retention, Clinical Study [Publication Type], Urinary Bladder, Urinary Incontinence, Catheterization

## Abstract

**Objectives:**

To understand the clinical relationship between lower limbs functions and the recovery of spontaneous voiding after an acute urinary retention (AUR) in older patients admitted to hospitals for non-urological causes using clinical parameters.

**Materials and Methods:**

56 adult patients (32 men; mean age: 77.9 ± 8.3 and 24 women; mean age 82.1 ± 4.6) with AUR were prospectively followed with validated Physical Performance Mobility Exam (PPME) instrument to evaluate the relationship between the recovery of mobility capacity and spontaneous voiding. After a short period of permanent bladder drainage patients started CIC along evaluation by PPME during hospitalization and at 7, 15, 30 60, 90, and 180 days of discharge. Mann-Whitney U, chi-square test and ANOVA tests were used.

**Results:**

All patients were hospitalized for at least 15 days (Median 26.3 ± 4.1 days). Progressive improvement on mobility scale measured by PPME was observed after leaving ICU and along the initial 7 days of hospitalization but with a deterioration if hospitalization extends beyond 15 days (p<0.03). Prolonged hospital stay impairs mobility in all domains (p<0.05) except step-up and transfer skills (p<0.02) although a recovery rate on spontaneous voiding persistented. Restoration of spontaneous voiding was accompanied by improvement on mobility scale (p<0.02). Recovery of spontaneous voiding was markedly observed after discharging the hospital. All patients recovered spontaneous voiding until 6 months of follow-up.

**Conclusions:**

Recovery to spontaneous voiding after acute urinary retention in the hospital setting may be anticipated by evaluation of lower limbs function measured by validated instruments.

## Introduction

Diminished independence of hospitalized older people is associated with an increased risk of transfer to nursing homes, mortality and healthcare costs after hospital discharge (1).

Patients resting or immobilized in hospital beds are a common clinical situation in different disease states. Frail older patients more commonly experience bed restriction during their recovery from the primary disease due to established generalized weakness or loss of consciousness (2).

In many circumstances, comorbidities are the decisive contributors to frailty and the inability to void, leading to secondary acute urinary retention (AUR) and permanent bladder drainage. Restoration of spontaneous voiding is a stressful event in the hospital setting because it can hardly be predicted, extending the period of undesired hospitalization even after general clinical improvement.

We hypothesized that diminished leg activity due to bed restriction secondarily predisposes patients to AUR resulting from the impaired neurogenic input from the neural sacral roots and poorly understood neurotrophic factors. Therefore, restoration of spontaneous voiding after a period of bed rest may be parallel to the functional recovery of the lower limbs.

## Material and Methods

During an 11-year period (1999 to 2010), 63 consecutive patients were prospectively enrolled from a general internal medicine ward after a urological evaluation was requested by the admitting Intensive Care Unit (ICU) team due to a palpable suprapubic mass and/or pain, absence of urine output or paradoxical incontinence. Seven cases were unsuitable for completion of the entire study period (3 cases died during hospitalization; 3 cases decided to have permanent urethral catheterization and 1 case was permanently disabled), leaving 56 consecutive patients (32 men; mean age: 77.9±8.3 and 24 women; mean age 82.1±4.6) that were closely followed according to the established protocol after an urological evaluation was requested due to AUR in an adult general hospital.

Patients with chronic neurological conditions such as brain or spinal cord injuries, peripheral or post-operative neurological diseases, endstage oncological disease, alcoholism, psychiatric disorders or drug abuse as well as acute or chronic use of cholinergic or anticholinergic agents were not accrued for the study.

Patients were also excluded if they could not follow simple commands and cooperate with the protocol, were delirious, had unstable fractures, had unstable cardio-respiratory status; had paraplegia or a major limb amputation, were demented or presented an impaired Folstein Mini-Mental State Examination (MMSE - score <24).

All patients or their responsible party reported they were able to walk across a small room without the assistance of another person prior to the hospital admission and were able to void normally with no previous urological evaluation or surgery to treat any dysfunctional voiding.

The inability to void was observed by the initial attending physician who requested a urological evaluation after hospital admission for various clinical reasons, none of them primarily related to urological problems (Table-1).

**Table 1:** Demographics of patients and main clinical reason for the admittance to the hospital before having urological evaluation and diagnosed as acute urinary retention after hospitalization.

Reason for Hospitalization	Men	Women
Age (±SD)	77.9 ± 8.3	82.1 ± 4.6
Protracted abdominal operation	7	9
Diabetes mellitus decompensation	6	4
Myocardial infarction	6	2
Pneumonia	4	3
Hip fracture	4	3
Diminished consciousness at home (mainly dehydration)	2	3
Acute Stroke	2	1

After careful urological evaluation, if the patient was unconscious or impaired to initiate a clean intermittent catheterization (CIC) regimen, they were spared from the study until they recovered and cooperated with the maneuvers described below to try to reestablish spontaneous voiding.

Patients first admitted to the ICU and who developed AUR received permanent urethral catheter until they could understand, cooperate and leave the bed. If the patient stayed in the ICU but was restricted to the bed, the protocol was postponed until they were discharged to the ward where the spontaneous voiding trial could be initiated, based on improvements in the general clinical condition as well as on consciousness and the ability to leave the bed.

If they were initially evaluated at the ward due to AUR and were not cognitively impaired or restricted to the bed, they had a permanent urethral catheter for 3 days after restoration of spontaneous voiding was attempted. If spontaneous voiding was restored post-void, residual and AUR were ascertained for 2 days before the patient was discharged. If the patient could not void after 3 days on a permanent urethral catheter, then the CIC regimen was initiated on the 4^th^ day every 4–6 hours or on demand until the patient could void spontaneously, maintaining CIC for an additional 2 days after reestablishing spontaneous voiding to ascertain that post-void residue was consistently <100mL. If the patient was discharged from the hospital during this period, instructions were maintained for further urological follow-up. An auto-catheterization regimen was strongly stimulated, but a third-person caregiver was also instructed on the urethral catheterization in case of necessity or lack of autonomy.

**Figure 1:**
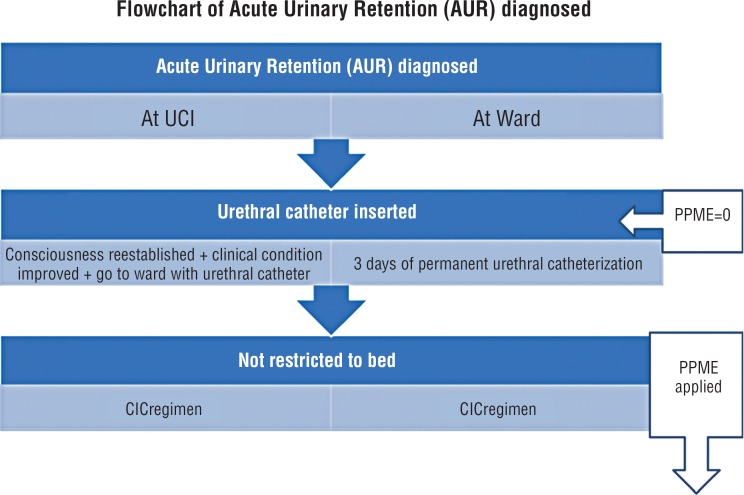
Period of hospitalization and discharge rate of 56 patients admitted for acute conditions.

At the time of catheterization and inclusion in the study, the Physical Performance Mobility Exam (PPME) was scored as zero. Subsequent PPME evaluations were performed during periodical urological office or home visits at 7, 15, 30 60, 90, and 180 days after discharge (Figure-1).

PPME was used by assistant personnel as a tool to objectively characterize the mobility scale (3) on a weekly basis until hospital discharge and on programmed domiciliary or office visits as described and graphed. PPME was also applied in a retrospective manner by the familiar or third-party caregiver if the patient was admitted unconsciously.

The standard of care for the physical rehabilitation program included respiratory exercises and motor activities coached by a licensed physiotherapist twice a day during the hospitalization period (morning and evening).

Upon hospital discharge, patients were required to be able to stand and walk independently. No further coached physical therapy by a physiotherapist was carried out at home, although basic exercises and walking at home at least 4 times a day was strongly reinforced.

Differences among the assessments of the physical domains throughout the study were performed using multivariate analysis of variance (ANOVA). Univariate analyses from the general linear model were also reported. Between groups of physical domains of PPME and in comparison to the rate of recovery of spontaneous voiding, factorial ANOVA for continuous measures, Mann-Whitney U and chi-square tests were used.

Medians were used to construct the presented graphics with standard deviation removed to facilitate graphical viewing. The IRB of the two involved hospitals approved the study.

## Results

A total of 48 cases were evaluated at the ward, whereas 8 were evaluated at ICU. Four patients were not unconscious, but they were cognitively impaired, impeding any prompt attempt to restore spontaneous voiding or start CIC. These cases were kept on permanent urethral Foley catheter until they could cope with the maneuvers (7, 7, 9 and 12 days, respectively).

Four patients presented overflow incontinence.

**Figure 2:**
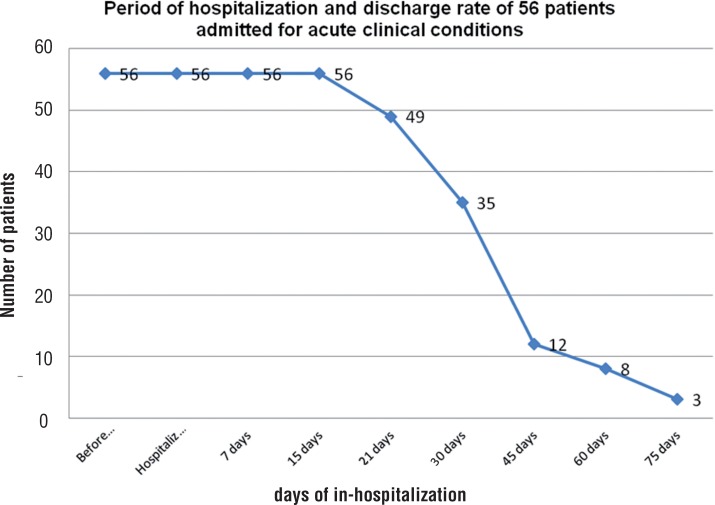
Flowchart of Acute urinary Retention in 56 patients admitted to hospital due to other than urological condition.

All 56 studied patients were hospitalized for at least 15 days with a decreasing number of cases, extending the hospitalization period beyond 30 days, as depicted in Figure-2. The median time of hospitalization was 26.3 days (SD±4.1 days).

Four patients promptly reestablished spontaneous voiding after the catheter was removed, but recurred on the AUR during their stay at the hospital. They were recorded as initiating the protocol from the point at which they started CIC.

**Figure 3:**
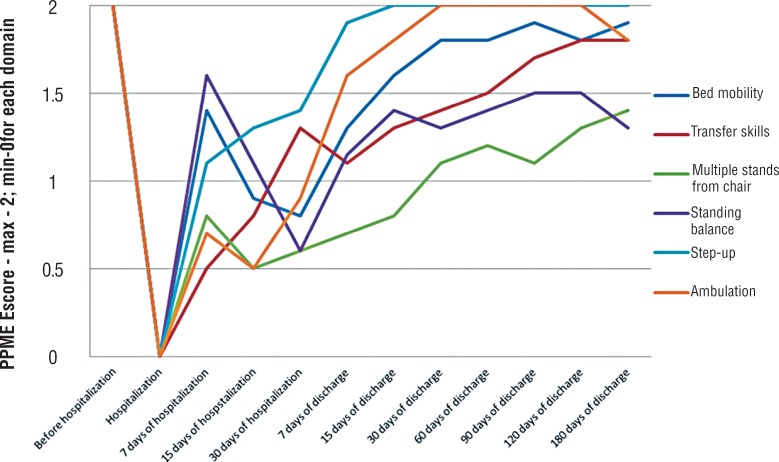
Evolution of PPME by each domain along 180 days of study.

Figure-3 shows that there were 3 points of inflexion regarding the evolution of mobility. The first inflexion concerns the significant improvement in the mobility scores at 7 days of hospitalization, possibly paralleling clinical improvement (p<0.002).

However, additional time in the hospital further impaired the mobility index when measured as the capacity to move on the bed, stand from a chair, equilibrate and walk (p<0.03). It is remarkable that those parameters worsened further until 30 days of hospitalization when the patient remained in the hospital (p<0.02).

Seven days after hospital discharge, a continuous trend of improvement in the mobility indexes was followed by a steady recovery in the capacity to void spontaneously (p<0.03).

Figure-3 also showed that some physical capacities improved faster than others with some improvement in a steady fashion, whereas others, such as bed mobility, standing balance and ambulation, obeyed the previously mentioned impairment if hospitalization extended beyond 7 days, further improving after hospital discharge (p<0.05).

**Figure 4:**
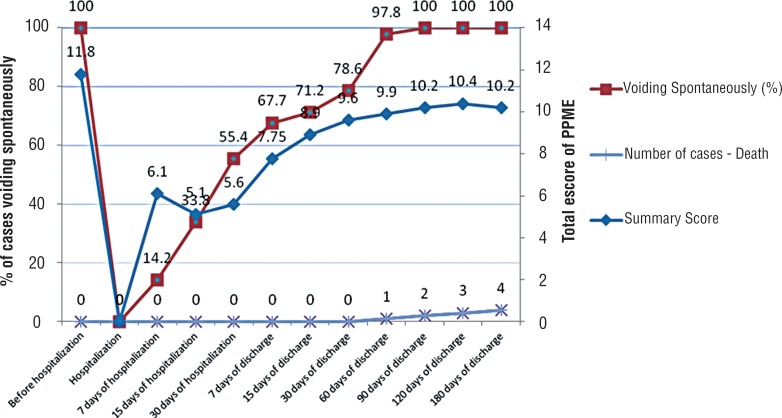
Evolution of recovery for spontaneous voiding matched to PPME index.

Notably, after urinary retention was established and diagnosed, spontaneous voiding was recovered and paralleled the improvement of the PPME index, although an initial decrease in the average physical ability was measured in the initial 15 days of hospitalization, which did not impact the recovery of voiding (Figure-4).

It was also noted that 4 patients died during the 180-day follow-up period, although all patients were spontaneously voiding 90 days after hospital discharge. The causes of death were not studied.

## Discussion

Half of all hospital beds are occupied by people >65 years of age, and it is expected that this demand will increase to 70% by 2050 (4).

Geriatric syndromes, including dementia, polypharmacy, falls, incontinence, pressure ulcers, sensory impairment, and malnutrition (5), are clinical representations of multifactorial etiologies and are associated with an increased risk for adverse outcomes. Functional limitations limit survival and decisively contribute to frailty (6).

Frail individuals often have immobility, gait abnormalities, muscle weakness and poor balance (7). They may have difficulty performing activities of daily living (ADL) and instrumental activities of daily living (IADLs), which are critical to maintaining function.

The functional independence of older people is an indicator of their health status, and diminished independence in hospitalized older people is associated with an increased risk of care burden, mortality and healthcare costs after discharge (1, 8). Whatever the cause, any hospitalization event increases morbidity due to necessary or circumstantial immobilization in bed, promoting patient functional decline during the hospitalization period (9).

Among the problems acquired during immobilization, urinary retention is an undesirable side effect. It is difficult to manage and shows an unpredictable pattern of recovery, frequently extending the period of hospitalization and secondarily aggravating frailty.

Furthermore, the prolonged or necessary use of a urethral catheter reflects the need for third-party assistance, increased costs or the family's insecurity in taking the patient home.

Validated instruments for the measurement of the clinometric properties of mobility in hospitalized older acute medical patients may present floor or ceiling effects.

In this regard, Timed Up and Go (TUG) (10), Functional Ambulation Classification (FAC) (11) and the Barthel Index (BI) (12) were focused on mobility, but they presented limitations in acutely hospitalized patients.

Although PPME was not tested in older acute medical patients, it captured a minimal clinically important difference, presented none of the previously mentioned restrictions, was not greatly influenced by mood or mental status, reflected only the patient's dependency on mobility, and was performed easily and quickly by staff with no special training (13).

The reasons that urinary retention developed in frail older adults cannot be ascertained for certain as it may result from a wide range of physical and cognitive abilities (14, 15). As we often observe AUR in the hospital setting with no urological cause, we hypothesized that resting in bed might result in low neuronal output activities from the same sacral roots as the bladder and lower limbs, which can be clinically measured with tasks for the lower limbs.

Transtibial Electrical Nerve Stimulation (TENS) produced an inhibitory effect on overactive bladder, revealing the linkage between lower limb nerves and pelvic functions (16). Therefore, resuming spontaneous voiding could be measured by lower limb refunctionalization, which may reflect the functionality of the internal pelvis. Our study confirms this assertion, showing a clear relationship between both functions. The empirical observation of AUR as a frequent but transient complication after hernioplasty with no clear reason reinforces this assumption (17).

Hip fracture surgery is a comparable clinical situation. Patients with bed rest present a higher incidence of AUR, which can be reversed by earlier patient mobilization and intermittent catheterization with a return to spontaneous voiding in 5.1 days compared to 9.4 if an indwelling catheter was inserted (18); all patient's voiding was fully restored within 3 months (19).

This may explain why after 7 days of hospitalization, patients are impaired in their mobility scale, although the rate of recovery of spontaneous voiding improves, revealing that the CIC regimen is a powerful stimulus in restoring bladder function.

We assumed that voiding and PPME was normal based on retrospective interviews with the patient or their caregiver as the PPME score was at its maximum before hospitalization. Although we recognize that this could be a flaw in our assumptions, Covinsky et al. (20) also observed that retrospective evaluation is reliable. The main factor evaluated in this study was secondary AUR and its evolution as it relates to the mobility index ranked by PPME.

As previously studied, interventional measures in acutely ill patients to improve mobility scale reduce the length of the hospital stay (12), and all domains of these parameters were clearly improved in staggered fashion after the patients were discharged home.

One limitation may be the fact that urinary residual volume was not measured, but some authors demonstrated no relationship between PVR and improvement in mobility in a rehabilitation ward for older adults (21).

As previously mentioned, hospitalization may decrease lower body function as early as the second day (1), which could also be seen in our population from the severe decrease in mobilization and voiding capacity measured by PPME.

The recovery of lower limb function as measured by the various domains of PPME returned staggeringly and progressively on the days immediately after departure from the ICU, but with recognized impairment if the hospitalization extended beyond 7 days. This is in accordance with the results of studies showing lower body function as a basic indicator of health and recovery in older people (22), which tends to normalize by discharge (23), as was observed at home.

Patients were discharged into the community, abiding by the country's cultural and socio-economic realities. We do recognize that discharged patients who require care-givers are frequently sent to nursing homes, but one of the strengths of our study is the observational nature of spontaneous recovery in home settings with few interventions because discharge from hospitals rarely involves nursing homes as it does in the United States. In this regard, our study showed new improvement in mobility indexes after 7 days at home in a continuum of the recovery of spontaneous voiding, possibly suggesting that aside from the greater clinical improvement in the primary disease, returning to the familiar environment may contribute to more frequent and a wider amplitude of movements than those of the restrictive hospital environment. We also observed only a partial recovery of the lower limbs, confirming the results of other studies in which 30% of patients treated for acute illness lost the ability to perform ADL compared to pre-admission levels (8). This observation highlights frailty, as demonstrated by the death rate over 180 days, reaffirming that lower mobility scores at baseline tests predict further aggravation of disability (24). The major finding of this study was that leg power measured by a validated instrument is a significant predictor of physical performance (25).

Despite the importance of our results, our study has limitations worthy of discussion as the relative heterogeneity of the studied population limits the generalizability of the results.

## Conclusions

Measuring the recovery of leg function by a simple validated instrument can reflect the recovery rate of spontaneous voiding after AUR due to non-urological causes. We hypothesize that there may be an overlap between the neural sacral roots responsible for bladder function and lower limb functions.
